# A feature-engineered dataset of benign and phishing URLs for machine learning and large language models evaluation

**DOI:** 10.1016/j.dib.2025.112162

**Published:** 2025-10-10

**Authors:** Dam Minh Linh, Tran Cong Hung

**Affiliations:** a Information Security Technology Laboratory, and Faculty of Information Technology, Posts and Telecommunications Institute of Technology (PTIT), Ho Chi Minh, Vietnam; b Faculty of Engineering and Computer Science, Saigon International University (SIU), Ho Chi Minh, Vietnam

**Keywords:** Artificial intelligence (AI), Cybersecurity, Data science, Feature-engineered dataset, Large language models (LLMs), Machine learning (ML), Natural language processing (NLP), URL classification

## Abstract

Phishing websites remain a major cybersecurity threat, yet the availability of balanced and feature-rich datasets for evaluating detection models is still limited. While machine learning (ML) and large language models (LLMs) have shown strong potential in URL-based classification, most public datasets provide raw URLs without feature engineering, making reproducibility and fair comparison across models difficult. To address this gap, we present a curated dataset of 111,660 URLs, consisting of 100,000 benign samples (label 0) and 11,660 phishing samples (label 1). Each URL entry is enriched with 22 numerical lexical and structural features (e.g., URL length, domain length, digit ratio, entropy, HTTPS usage). Additionally, three string reference columns (URL, domain, TLD) are preserved for interpretability, and one label column (0 = benign, 1 = phishing), totaling 26 columns. To demonstrate its utility, we evaluate two baseline approaches: a Random Forest (RF) classifier using handcrafted features, and a MiniLM embedding model with Logistic Regression (LR). Both achieved accuracy above 96 % and ROC AUC scores exceeding 0.99 across training, validation, and test splits. This dataset represents an important step toward building reproducible and comparable benchmarks for phishing detection, bridging traditional ML and LLM-based approaches, and supporting future research on adversarial robustness and scalable security models.

Specifications Table

**Table:** 

Subject	Computer Science
Specific subject area	Artificial Intelligence, Cybersecurity, Phishing Detection
Type of data	Table in a csv or xlsx file
Data collection	The dataset comprises 111,660 URLs, including 100,000 benign samples obtained from trusted domains (e.g., educational, governmental, Alexa Top Sites) via a curated Zenodo repository [1], and 11,660 phishing samples collected from PhishTank [2] between November 2024 and September 2025. Each entry was processed to extract 26 lexical and structural features, cleaned to remove duplicates and inconsistencies, and split into train/validation/test subsets (75/10/15). Baseline models (RF and MiniLM + LR) were applied to validate dataset usability.
Data source location	Ho Chi Minh City, Vietnam (Posts and Telecommunications Institute of Technology).
Data accessibility	Repository name: URL-Phish: A Feature-Engineered Dataset for Phishing Detection Data identification number: doi:10.17632/65z9twcx3r.1 Direct URL to data: https://data.mendeley.com/datasets/65z9twcx3r/1 Data format: CSV (comma-separated values). Each row corresponds to one URL entry, with 25 feature columns and one label column.
Related research article	Draft title: *A Feature-Engineered Dataset of Benign and Phishing URLs for Machine Learning and LLM Evaluation*. Submitted to Data in Brief.

## 1 Significance of the Dataset

• This dataset provides a large-scale, feature-engineered collection of benign and phishing URLs, enabling reproducible and fair evaluation of detection models.
• Although imbalanced (100,000 benign vs. 11,660 phishing samples), the dataset reflects real-world phishing scenarios, making it valuable for developing robust classifiers under skewed class distributions.
• It includes 22 numerical features, 3 reference columns, and 1 label column (total 26 columns), supporting both traditional ML approaches and modern LLM-based methods.
• Researchers in cybersecurity, NLP, and AI can use this dataset to benchmark algorithms for phishing detection, adversarial robustness, and explainable AI.
• Policymakers, educators, and industry professionals can leverage this dataset for cybersecurity training, awareness programs, and deployment-ready detection systems.

## 2 Background

Phishing continues to escalate as a major cybersecurity threat. In the first quarter of 2025, there were 1003,924 phishing attacks, the largest number since late 2023. The financial and online payment sectors were the most targeted, accounting for 30.9 % of all attacks, while business email compromise wire transfer fraud rose by 33 % compared to the previous quarter. These statistics highlight the growing sophistication of phishing tactics and underscore the urgent need for reliable datasets to benchmark and evaluate detection models [3]. According to the FBI’s Internet Crime Complaint Center, Americans lost a record 16.6 billion USD to cyber-enabled fraud and scams in 2024—an increase of 33 % over 2023—based on >859,000 complaints submitted by victims [4].

Existing phishing datasets still present notable limitations in terms of scale and timeliness. For example, the PhishStorm dataset [5] contains only 96,018 URLs (48,009 benign and 48,009 phishing). Similarly, the study in [6] used approximately 10,000 URLs, combining both URL and webpage content for Transformer-based training, which makes fair comparison with URL-only approaches difficult. In addition, Buu et al. [7] proposed a fuzzy-calibrated transformer network for phishing URL detection, but the model was evaluated on the ISCX-URL2016 dataset, which includes only 35,000 benign URLs, 9000 phishing, 11,000 malware, and 12,000 spam URLs—an outdated and relatively small-scale dataset.

These limitations highlight the urgent need for a new phishing URL dataset that is large-scale, feature-rich, and easily accessible, in order to support fair benchmarking and reproducible evaluation in both ML and LLM research.

Therefore, a new large-scale and feature-engineered dataset is required to support reproducible, fair, and comprehensive evaluation of phishing URL detection methods. To demonstrate its utility, the proposed URL-Phish dataset is benchmarked using two complementary approaches: a traditional ML model [8] (Random Forest) trained on handcrafted lexical and structural features, and a lightweight large language model [9] (MiniLM) combined with LR for URL embedding classification.

Both methods are evaluated using standard metrics [[10], [11], [12]]—Accuracy, Precision, Recall, F1-score, ROC AUC, and Confusion Matrix analysis—across train, validation, and test splits, thereby validating the dataset’s effectiveness for diverse detection paradigms.

## 3 Dataset Description

The proposed dataset, termed URL-Phish, comprises a total of 111,660 URLs, including 100,000 benign samples (label = 0) and 11,660 phishing samples (label = 1). The dataset was curated through a systematic process, consisting of data collection, preprocessing, feature engineering, partitioning, and baseline verification.

(a) Data Sources.

– Benign subset (100,000 samples): Benign URLs were collected from *trusted domains*, including educational (.edu), governmental (.gov), and highly ranked commercial websites (e.g., Alexa Top Sites). To ensure reliability, this subset was derived from a publicly available Zenodo dataset [1], which provides curated URL lists registered with a DOI.
– Phishing subset (11,660 samples): Phishing URLs were obtained from community-driven repositories, primarily PhishTank [2], which aggregates and validates reports of malicious URLs. This subset covers the period from 2024 to 11–12 to 2025–09–24, thereby ensuring temporal diversity in phishing campaigns.

(b) Preprocessing.

All collected URLs underwent a standardized preprocessing pipeline:

– Duplicate removal: Eliminated redundant entries that appeared across multiple sources.
– Invalid entry exclusion: Removed URLs with missing values or incorrect structures.
− Normalization: Standardized character encoding (UTF-8) and converted all strings to lowercase for consistency.

(c) Feature Engineering.

Each URL was enriched with 26 lexical, structural, and metadata attributes to support ML and LLM-based classification tasks. The features include:

– Length-based attributes: total URL length, domain length, path length, and query length.
− Character distribution attributes: counts and ratios of letters, digits, and special characters, along with Shannon entropy.
– Structural attributes: number of subdomains and frequency of specific symbols (e.g., “/”, “=”, “?”, “-”, “_”, “&”, “.”).
– Protocol attribute: HTTPS usage (binary).
– Reference attributes: original URL string, extracted domain, and TLD (retained for interpretability but excluded from modeling).

A detailed description of these features is provided in Table 1.

**Table 1:** Description of the 26 columns in the phishing URL dataset, including 22 numerical features, 3 reference columns, and 1 label column.

No.	Feature	Type	Description
1	url_len	Integer	Total length of the URL string.
2	dom_len	Integer	Length of the domain part.
3	is_ip	Binary	1 if the domain is an IP address, else 0.
4	tld_len	Integer	Length of the top-level domain (TLD).
5	subdom_cnt	Integer	Number of subdomains.
6	letter_cnt	Integer	Count of alphabetic characters.
7	digit_cnt	Integer	Count of numeric characters.
8	special_cnt	Integer	Count of special characters.
9	eq_cnt	Integer	Number of equal signs = in the URL.
10	qm_cnt	Integer	Number of question marks ?.
11	amp_cnt	Integer	Number of ampersands &.
12	dot_cnt	Integer	Number of dots ..
13	dash_cnt	Integer	Number of dashes -.
14	under_cnt	Integer	Number of underscores _.
15	letter_ratio	Float	Ratio of letters to total URL length.
16	digit_ratio	Float	Ratio of digits to total URL length.
17	spec_ratio	Float	Ratio of special characters to total URL length.
18	is_https	Binary	1 if HTTPS protocol is used, else 0.
19	slash_cnt	Integer	Number of slashes /.
20	entropy	Float	Shannon entropy of the URL string (higher = more randomness).
21	path_len	Integer	Length of the URL path.
22	query_len	Integer	Length of the query string.
23	url	String	Original full URL (kept for reference, not used in modeling).
24	dom	String	Extracted domain name.
25	tld	String	Extracted top-level domain.
26	label	Binary	0 = benign, 1 = phishing.

### 4 Proposed method and experimental setup

For reproducibility, the dataset was randomly partitioned into three subsets: 75 % for training, 10 % for validation, and 15 % for testing. This partitioning strategy ensures a fair evaluation of model generalization while preventing overfitting.

To further demonstrate the usability of the dataset, two baseline classifiers were implemented as part of the experimental setup:

– RF trained on handcrafted lexical and structural features.
– MiniLM embeddings combined with LR for semantic URL classification.

**Fig. 1:**
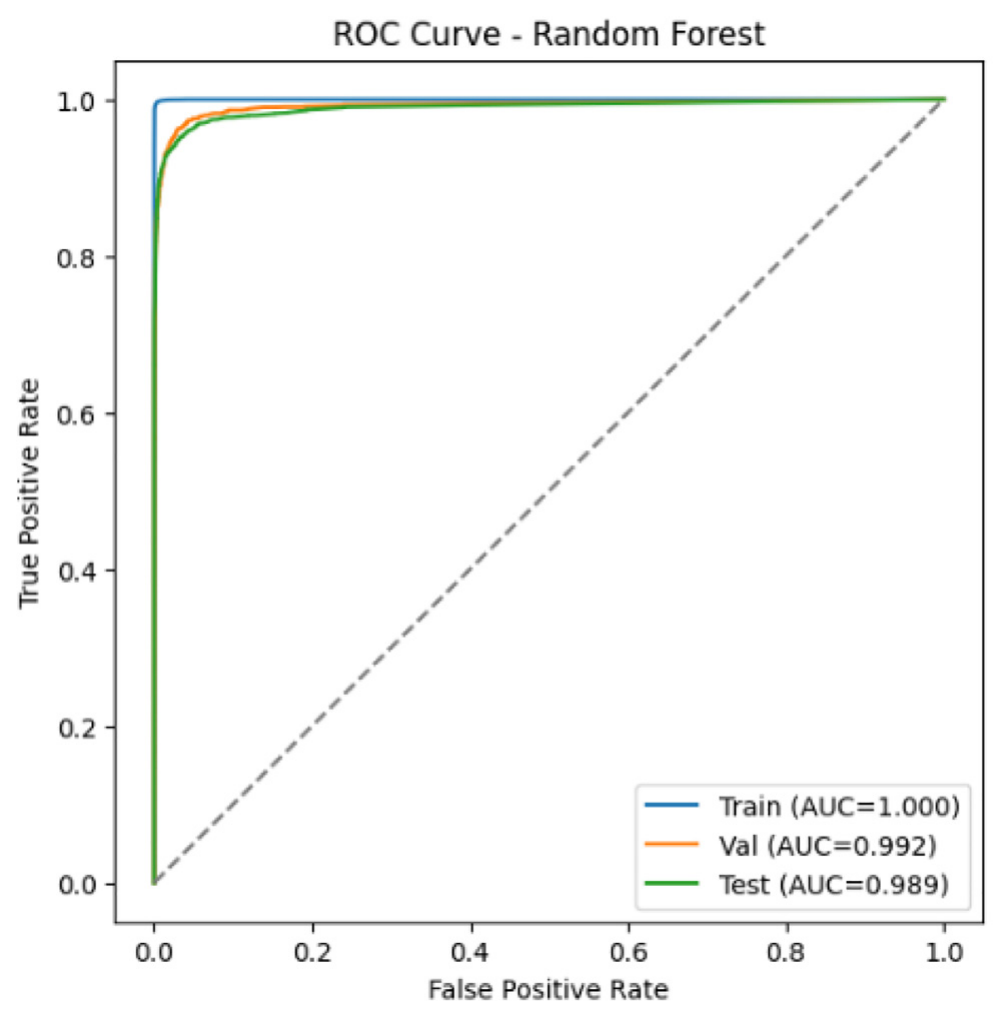
ROC Curve – RF.

**Fig. 2:**
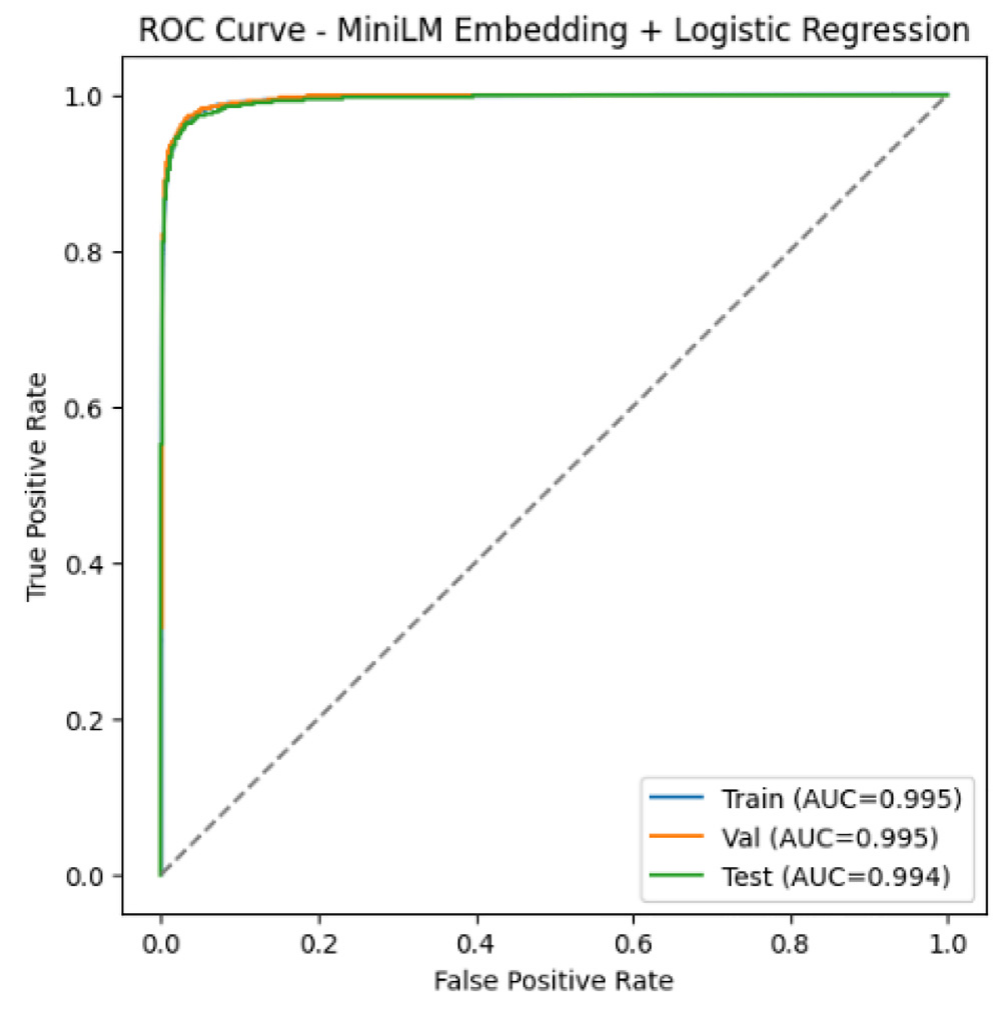
ROC Curve – MiniLM + LR.

Fig. 1, Fig. 2 present the ROC curves of the baseline classifiers evaluated on the proposed dataset. The RF attained AUC scores of 100.0 % on the training set, 99.2 % on the validation set, and 98.9 % on the test set. The MiniLM + LR model achieved 99.5 % on the training and validation sets and 99.4 % on the test set. Both classifiers consistently maintained performance above 98 % AUC, thereby demonstrating the robustness of the dataset and confirming the reliability of the baseline models for phishing URL detection.

**Fig. 3:**
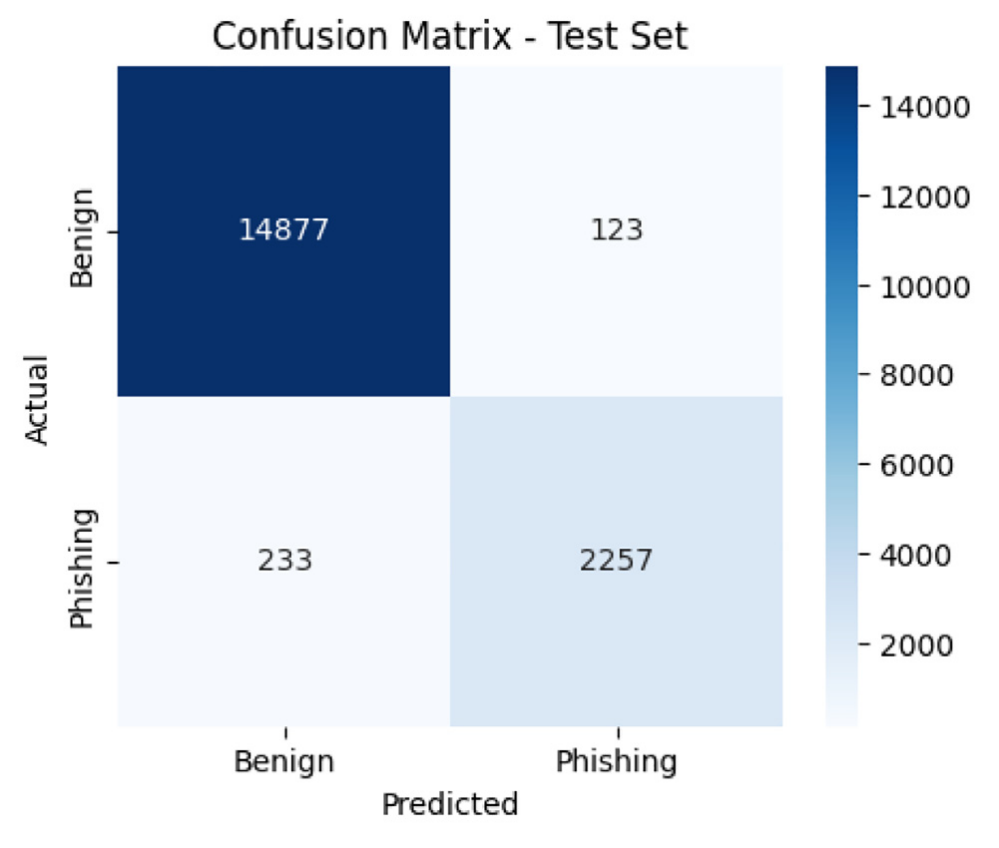
Confusion matrix of the RF classifier on the test set.

**Fig. 4:**
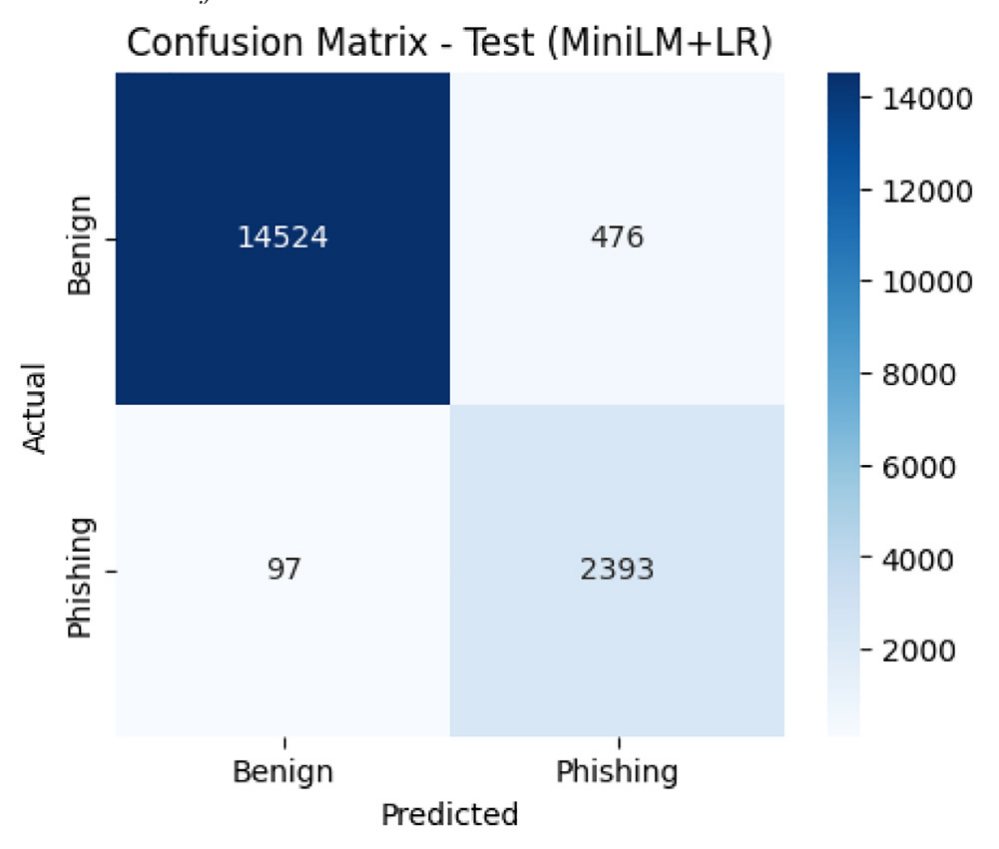
Confusion matrix of the MiniLM + LR classifier on the test set.

Fig. 3, Fig. 4 present the confusion matrices of the baseline classifiers evaluated on the test set. The RF correctly classified 14,877 benign and 2257 phishing URLs, with 123 benign misclassified as phishing and 233 phishing misclassified as benign. In contrast, the MiniLM + LR model identified 14,524 benign and 2393 phishing URLs correctly, while misclassifying 476 benign and 97 phishing URLs. These results indicate that RF slightly favors benign detection, whereas MiniLM + LR yields more balanced performance across classes.

**Fig. 5:**
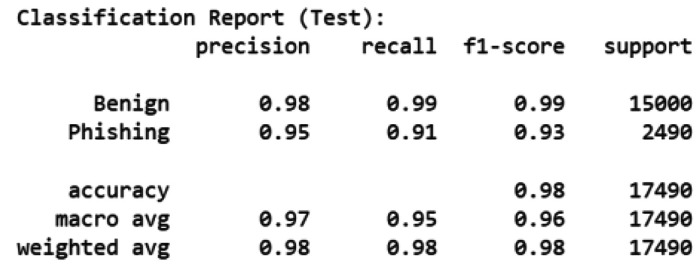
Classification report of RF on the test set.

**Fig. 6:**
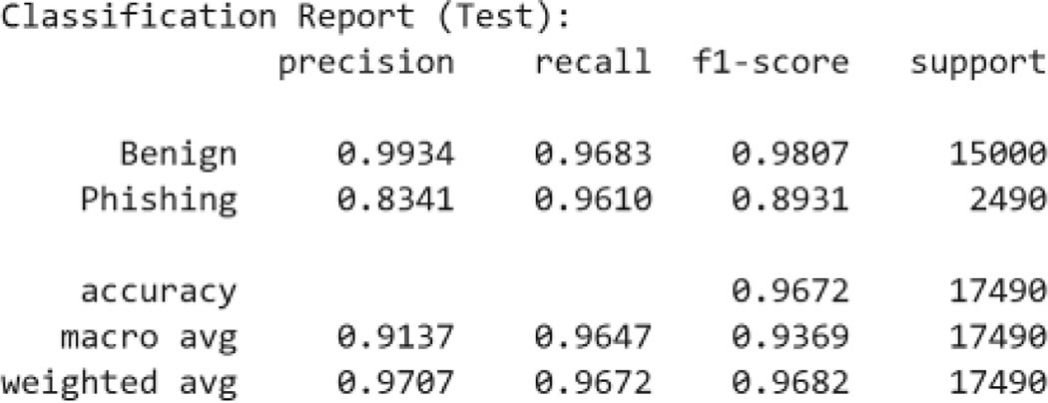
Classification report of MiniLM + LR on the test set.

Fig. 5, Fig. 6 summarize the classification reports of the baseline classifiers on the test set. RF achieved higher precision and F1-scores for both benign (0.98–0.99) and phishing (0.95–0.93) classes, leading to an overall accuracy of 98 %. In contrast, MiniLM + LR yielded slightly lower phishing precision (0.83) but comparable recall (0.96), resulting in an overall accuracy of 96.7 %.

**Fig. 7:**
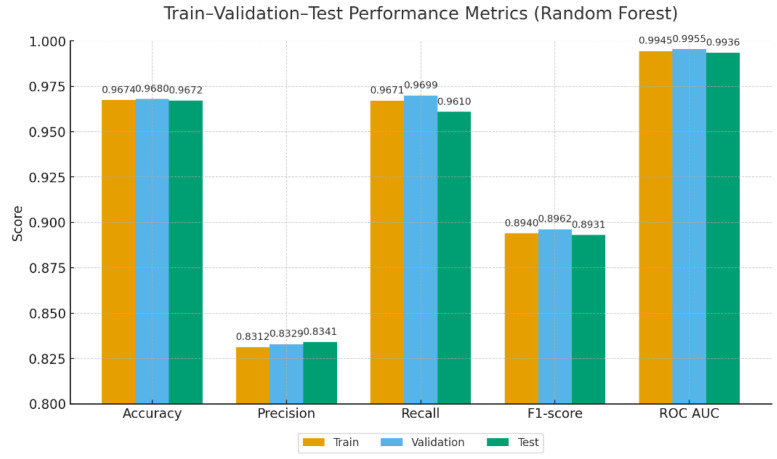
Train–validation–test performance metrics of Random Forest.

**Fig. 8:**
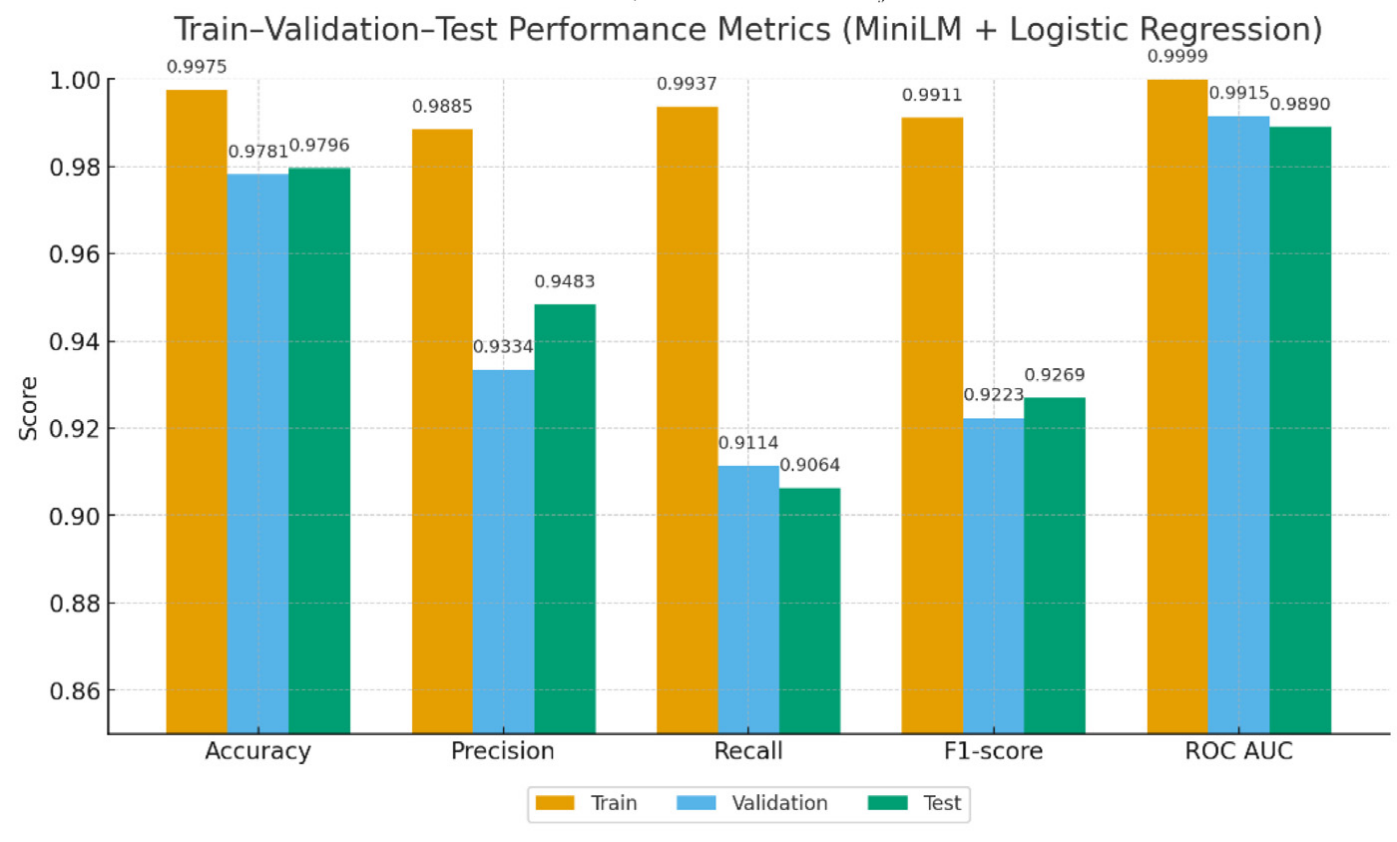
Train–validation–test performance metrics of MiniLM + LR.

Fig. 7, Fig. 8 summarize the performance metrics of the baseline classifiers across the training, validation, and test sets. RF achieved balanced accuracy (96.7–96.8 %) with high ROC AUC scores (99.4–99.6 %) but relatively lower precision (∼83 %). In contrast, MiniLM + LR exhibited stronger precision (93–98 %) and F1-scores (∼92–93 %), while maintaining overall accuracy close to 97 % and ROC AUC above 98.9 %.

The experimental results demonstrate that this research does not merely introduce a new and reliable dataset, but also provides comprehensive validation of its usability. By employing baseline models such as RF and MiniLM + LR, the proposed dataset was rigorously evaluated across multiple quantitative metrics, including accuracy, precision, recall, F1-score, and ROC AUC. These findings confirm both the robustness of the dataset and its potential to serve as a trustworthy benchmark for future phishing URL detection studies.

## Limitations

Although the proposed dataset demonstrates robustness and reliability, it also suffers from class imbalance, as the number of phishing samples is significantly lower than that of benign samples.

## Ethics Statement

This study uses only publicly available URL datasets, without any personal or sensitive information, for research and educational purposes in cybersecurity.

## Credit Author Statement

Dam Minh Linh: Conceptualization, Methodology, Software, Validation, Formal analysis, Investigation, Resources, Data Curation, Writing – Original Draft, Writing – Review & Editing, Visualization, Funding acquisition. Tran Cong Hung: Writing – Review & Editing, Supervision, Project administration, Funding acquisition.

## Acknowledgments

The authors sincerely thank the Editor-in-Chief, the reviewers, and the Associate Editor for their constructive and valuable feedback. This research was financially supported by the Posts and Telecommunications Institute of Technology (PTIT), Vietnam.

## Declaration of Competing Interest

The authors declare that they have no known competing financial interests or personal relationships that could have appeared to influence the work reported in this paper.

## Data Availability

Mendeley DataURL-Phish: A Feature-Engineered Dataset for Phishing Detection (Original data).
